# A single dose of radiation elicits comparable acute salivary gland injury to fractionated radiation

**DOI:** 10.1242/dmm.050733

**Published:** 2024-08-22

**Authors:** Amanda L. Johnson, Sonia S. Elder, John G. McKendrick, Lizi M. Hegarty, Ella Mercer, Elaine Emmerson

**Affiliations:** ^1^ The Centre for Regenerative Medicine, Institute for Regeneration and Repair, The University of Edinburgh, 4-5 Little France Drive, Edinburgh, EH16 4UU, UK; ^2^ The Centre for Inflammation Research, Institute for Regeneration and Repair, The University of Edinburgh, 4-5 Little France Drive, Edinburgh, EH16 4UU, UK

**Keywords:** Epithelia, Injury, Macrophages, Radiation, Salivary gland

## Abstract

The salivary glands are often damaged during head and neck cancer radiotherapy. This results in chronic dry mouth, which adversely affects quality of life and for which there is no long-term cure. Mouse models of salivary gland injury are routinely used in regenerative research. However, there is no clear consensus on the radiation regime required to cause injury. Here, we analysed three regimes of γ-irradiation of the submandibular salivary gland. Transcriptional analysis, immunofluorescence and flow cytometry was used to profile DNA damage, gland architecture and immune cell changes 3 days after single doses of 10 or 15 Gy or three doses of 5 Gy. Irrespective of the regime, radiation induced comparable levels of DNA damage, cell cycle arrest, loss of glandular architecture, increased pro-inflammatory cytokines and a reduction in tissue-resident macrophages, relative to those observed in non-irradiated submandibular glands. Given these data, coupled with the fact that repeated anaesthetic can negatively affect animal welfare and interfere with saliva secretion, we conclude that a single dose of 10 Gy irradiation is the most refined method of inducing acute salivary gland injury in a mouse model.

## Teaser

**Summary:** Using three regimes of γ-irradiation, a single 10 Gy dose was found to be the most refined method of inducing acute salivary gland injury in a mouse model to study the effects of head and neck cancer radiotherapy.

## INTRODUCTION

Therapeutic radiation remains a predominant treatment for targeting various cancers. Although recent advances in the delivery of radiation, such as intensity-modulated radiation therapy, aim to minimise off-target side effects, healthy tissues that lie within the therapeutic field often also receive high doses of radiation, leading to cellular damage and organ dysfunction (Bentzen, 2006). The salivary glands are often irreversibly damaged following radiotherapy for head and neck cancer, and the resulting salivary gland dysfunction results in xerostomia, or chronic dry mouth (Lechelt et al., 2018). These adverse side effects of radiation therapy substantially decrease the quality of life in survivors and there is currently no long-term regenerative therapy for this debilitating condition (Bentzen, 2006). Therefore, to better the quality of life in patients, the effects of radiation injury on salivary glands and the mechanisms by which the tissue repairs itself or fails to repair itself must be better understood.

Mice provide excellent preclinical models owing to their anatomical similarities to humans (Emmerson and Knox, 2018), coupled with their genetic tractability and the availability of transgenic strains. Research into salivary gland radiation injury routinely uses mice to model the tissue injury that patients undergoing radiotherapy experience. However, there is no clear consensus on the dose that is required to mirror the injury evident in patients. Patients with head and neck cancer generally receive between 50 and 70 Gy radiation (Bourhis et al., 2012; Thomson et al., 2020) targeted to the tumour, which is split over a 6 week period (termed fractionated radiotherapy). Although radiotherapy is often not purposefully targeted at the salivary glands, as the proportion of head and neck cancer that arises from salivary gland tissue is very low (<5%) (Lawal et al., 2015; Mezi et al., 2020), the salivary glands often inadvertently fall into the irradiation field and can experience doses of anywhere between 8 and 55 Gy (Hawkins et al., 2018; van Timmeren et al., 2021). This results in cell disorganisation, collagen deposition and adipocyte accumulation (Nam et al., 2016), as well as a loss of saliva production (Eisbruch et al., 2001; Henson et al., 1999) and/or a change in saliva consistency (Winter et al., 2021; Funegård et al., 1994; Makkonen et al., 1986). Acute hyposalivation occurs as early as 1 week after radiotherapy (Franzén et al., 1992), whereas longer-term changes are associated with acinar cell loss, ductal metaplasia and fibrosis (Sullivan et al., 2005) and lead to xerostomia in the majority of patients (Wijers et al., 2002). However, as human biopsy samples can only be accessed when a donor is already undergoing a surgical intervention, there is a dearth of data on the acute effects of radiation injury on human salivary gland tissue. As such, animal models are crucial to understand these early effects at the cellular level.

To date, various doses and regimes of radiation have been used in mouse models to mirror the human clinical situation, including single doses ranging from 1 Gy (Limesand et al., 2006; Humphries et al., 2006; Avila et al., 2009) to 15 Gy (Peng et al., 2020; Lombaert et al., 2008; Bralic et al., 2005; Takeda et al., 2003; Cotrim et al., 2007, 2005; Urek et al., 2005; Limesand et al., 2010), and fractionated doses ranging from 4 Gy (Limesand et al., 2010) to 30 Gy (Cotrim et al., 2007). Table 1 outlines the various radiation regimes used in the field and includes the type of radiation used in each study. However, it is widely known that different mouse strains or genetic backgrounds exhibit differences in their sensitivity to radiation in other organs (Jackson et al., 2010; Down and Yanch, 2010; Down et al., 1986; Dileto and Travis, 1996), and, as such, we have also included details of the genetic background used in all published studies. Regardless of the dose and type of irradiation injury, there are some commonalities reported across studies, including DNA damage, changes in acinar cells, fibrosis and loss of saliva production.

**Table 1.**
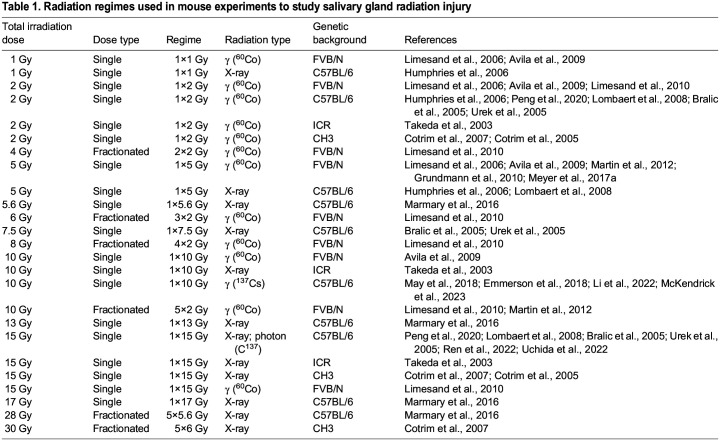
Radiation regimes used in mouse experiments to study salivary gland radiation injury

**Fig. 1.**
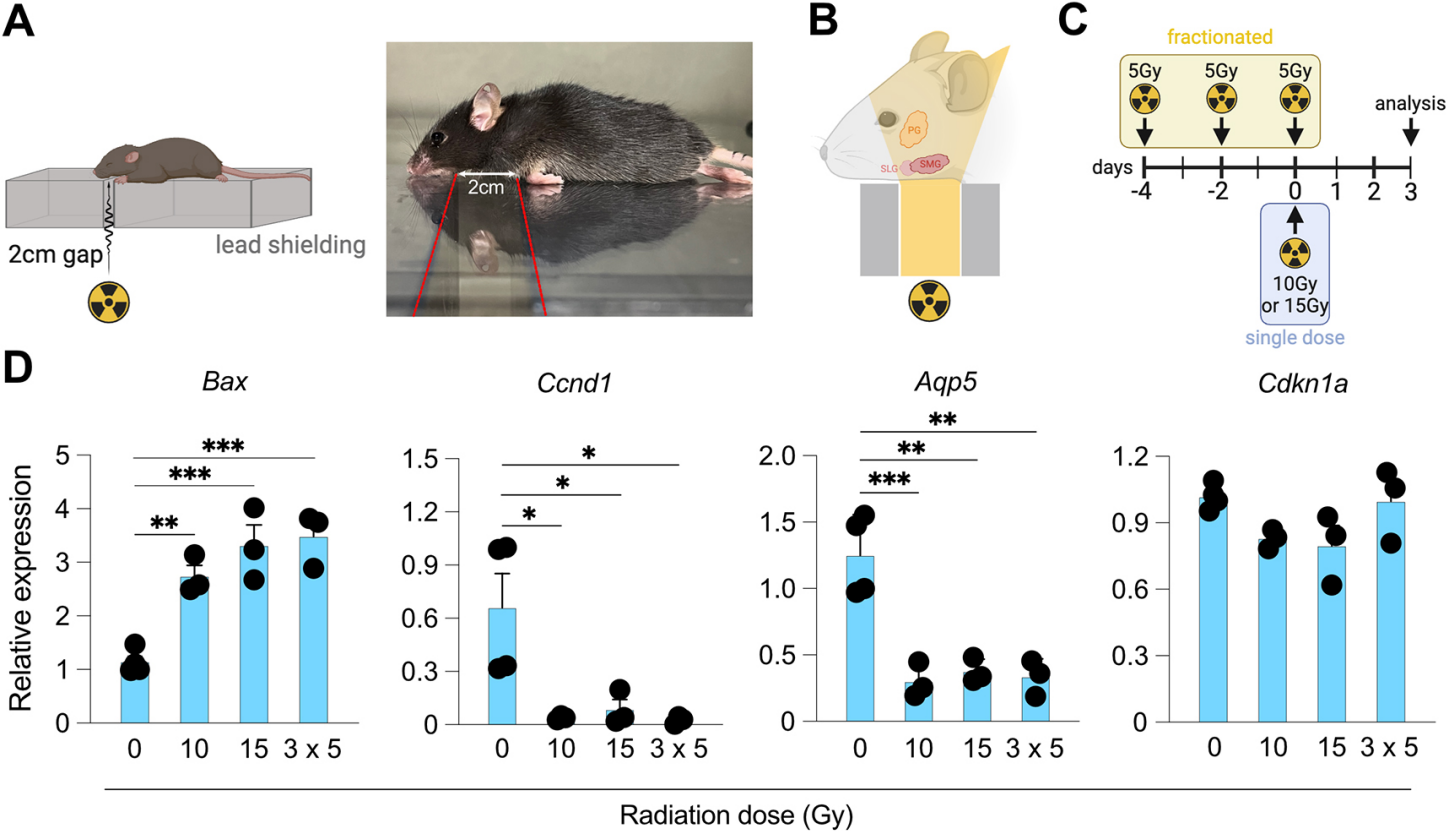
**Radiation induces cell cycle differences and functional changes in murine submandibular salivary gland.** (A) Schematic (left) and picture (right) of the experimental setup to expose murine salivary glands to ionising radiation. (B) Schematic to highlight the tissues and organs expected to be irradiated using this experimental setup. PG, parotid gland; SLG, sublingual gland; SMG, submandibular gland. (C) Experimental timeline showing the timing of fractionated (three 5 Gy doses over 5 days, 3×5 Gy) and single (10 or 15 Gy) doses of radiation with analysis at 3 days after the final dose. (D) qPCR analysis of *Bax*, *Ccnd1*, *Aqp5* and *Cdkn1a* mRNA in total SMG tissue at 3 days following radiation-induced injury. Data are normalised to mRNA levels in unirradiated (0 Gy) SMG tissues. Data are from three to four mice and error bars represent the s.d. *P<0.05; **P<0.01; ***P<0.001 (one-way ANOVA followed by Tukey’s *Q* post hoc test). The schematics in A-C were created with BioRender.com.

In this study, we compared three regimes of γ-irradiation, namely, single doses of 10 Gy and 15 Gy, and three doses of 5 Gy delivered over a period of 5 days (Fig. 1C), in order to ascertain and compare the levels of cellular damage elicited, focusing on the submandibular salivary gland (SMG), the best studied of the three major salivary glands in mice. We examined SMGs at 3 days after irradiation, as we have previously shown that, at this timepoint, we see significant cellular damage, disruption of the epithelial architecture and transcriptional changes associated with DNA damage and stress response to injury (Emmerson et al., 2018; McKendrick et al., 2023). Using transcriptional analysis, immunofluorescence staining and flow cytometry, we characterised epithelial and immune cell changes following the three different regimes of radiation injury, and we show that although there are subtle differences among different approaches, all regimes elicit comparable damage relative to that in unirradiated SMGs.

## RESULTS

We used a well-established model of targeted radiation to compare three regimes of γ-irradiation (Fig. 1A). This model exposes the three pairs of salivary glands, while protecting the nose and the body with lead (Fig. 1B). We delivered a single dose of either 10 or 15 Gy γ-irradiation, or three doses of 5 Gy irradiation over a period of 5 days (hereafter 3×5 Gy), followed by analysis 3 days after the final dose (Fig. 1C). We and others have previously demonstrated that radiation to either the SMG, sublingual gland or parotid gland causes transcriptional changes in the hours and days immediately after irradiation (4 and 8 h and 1-7 days post irradiation) that are indicative of DNA and cellular damage, including elevation in the expression of genes involved in apoptosis and cell cycle arrest [*Bax* and *Cdkn1a* (encoding p21)] (Avila et al., 2009, Emmerson et al., 2018, McKendrick et al., 2023) and a reduction in the expression of genes involved in cell cycle (*Mki67* and *Ccnd1*) and salivary gland function [*Aqp5* and *Mist1* (also known as *Bhlha15*)] (Emmerson et al., 2018; McKendrick et al., 2023). In this study, we first analysed gene expression changes at 3 days post irradiation in unirradiated SMGs (0 Gy) and SMGs exposed to 10 Gy, 15 Gy or 3×5 Gy. We found that all three radiation regimes elicited a similar and significant increase in the expression of *Bax*, a gene involved in the apoptotic and cell death pathway (Westphal et al., 2014), and a reduction in the expression of cyclin D1 (*Ccnd1*) and the water channel-encoding gene aquaporin 5 (*Aqp5*) (Fig. 1D). However, the expression of *Cdkn1a* was unchanged between unirradiated and irradiated SMGs (Fig. 1D).

**Fig. 2.**
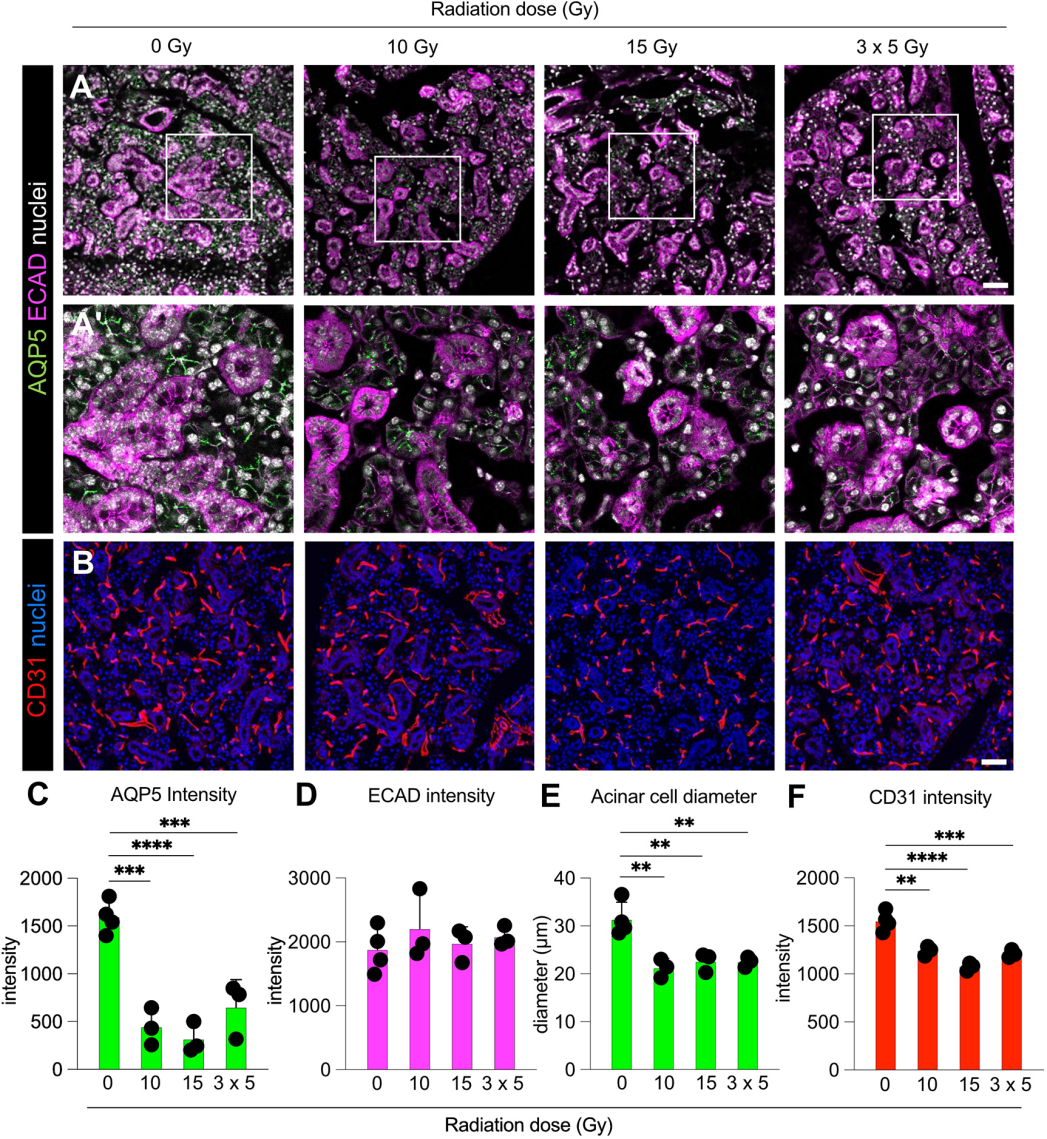
**Radiation injury alters epithelial architecture and vascularisation of murine SMG.** (A,B) Representative expression of aquaporin 5 (AQP5) and E-cadherin (ECAD) (A,Aʹ) or CD31 (B) in SMGs of uninjured mice or mice irradiated with single doses of 10 Gy or 15 Gy or multiple doses of 3×5 Gy and analysed 3 days after irradiation. A′ shows magnified views of the boxed areas in A. Scale bars: 100 µm. (C-F) Enumeration of AQP5 (C), ECAD (D) or CD31 (F) fluorescence intensity and measurement of acinar cell diameter (E) in mice exposed to 0 Gy, 10 Gy, 15 Gy or 3×5 Gy irradiation and analysed 3 days after irradiation. Data were obtained from three fields of view from non-sequential sections from three to four mice per group and error bars represent the s.d. **P<0.01; ***P<0.001; ****P<0.0001 (one-way ANOVA with Tukey's *Q* post hoc test).

We subsequently performed immunofluorescence analysis for markers of tissue architecture, DNA damage and cell cycle. We first analysed the epithelial architecture by staining for the epithelial marker E-cadherin (ECAD, encoded by *Cdh1*) and the acinar water channel aquaporin 5 (AQP5). AQP5 is necessary for water transfer and plays a major role in the salivary secretion process (Delporte and Steinfeld, 2006); thus, a loss of expression or aberrant localisation leads to salivary gland dysfunction. We found that, compared to the unirradiated control, the irradiated samples exhibited disruption to the tissue architecture, as previously reported (McKendrick et al., 2023), regardless of the radiation regime used (Fig. 2A,A′). We quantified AQP5 and ECAD fluorescence intensity (Fig. 2C,D) and found AQP5 levels to be in line with gene expression data (Fig. 1C). AQP5 fluorescence intensity was significantly reduced in all three radiation regimes (Fig. 2C), in line with the observed acinar cell disruption (Fig. 2A), whereas ECAD intensity was unchanged (Fig. 2A,D). This suggests that acinar cells are more sensitive than ductal epithelial cells to radiation at this early timepoint. Furthermore, we analysed acinar cell atrophy by measuring average acinar cell diameter as previously described (Lombaert et al., 2020). We found that acinar cell diameter was significantly reduced following irradiation, but it was not different among irradiation regimes (Fig. 2A,E). We also performed immunofluorescence analysis for CD31 (PECAM1), which is found on endothelial cells and is used as a marker of vasculature (Lertkiatmongkol et al., 2016). In line with previously published studies (Cotrim et al., 2007; McKendrick et al., 2023), we found that the fluorescence intensity of CD31 was modestly but significantly reduced following irradiation (Fig. 2B,F), suggesting that irradiation directly impacts vessel arrangement and architecture.

**Fig. 3.**
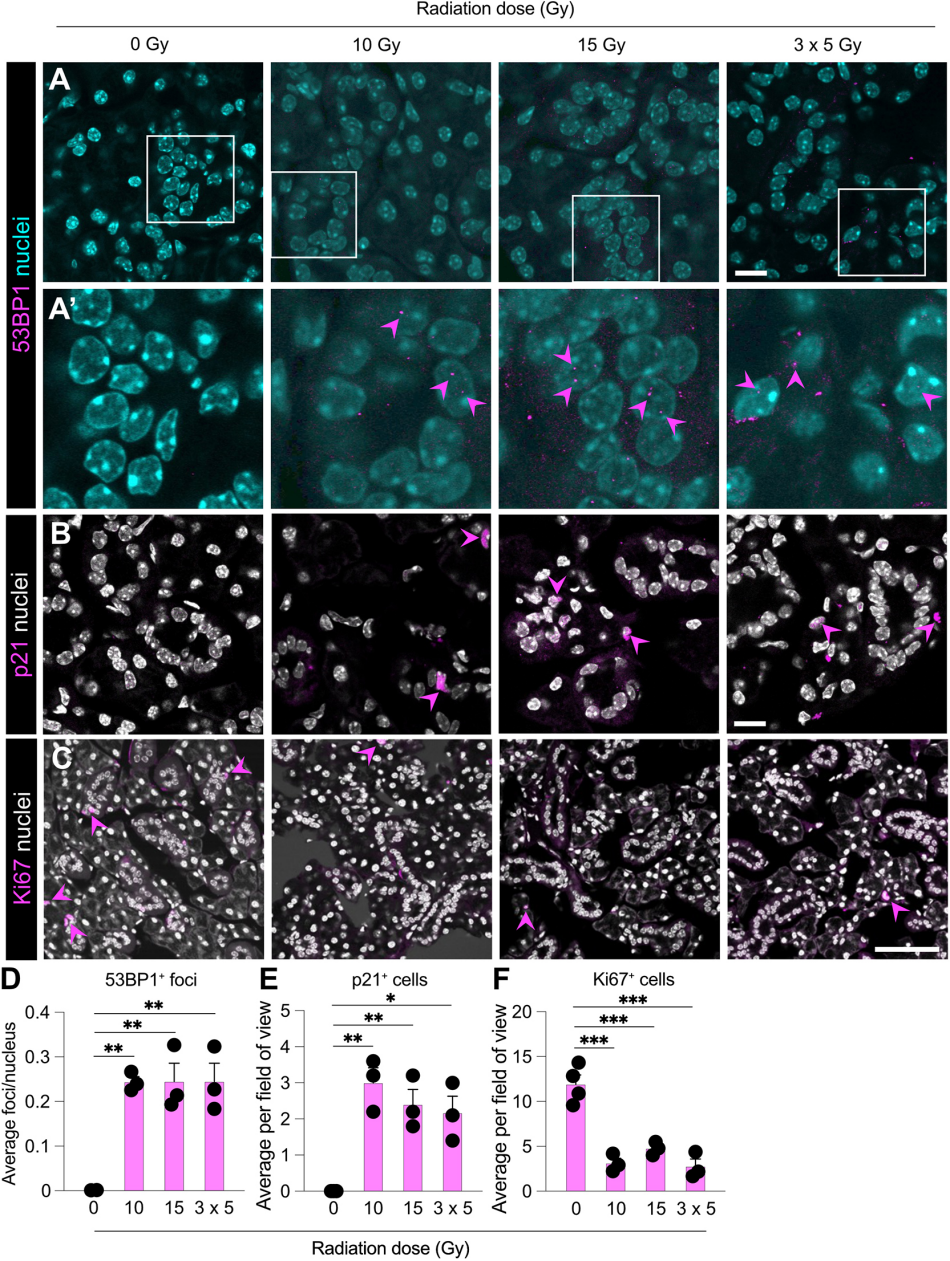
**Radiation induces DNA damage, cell cycle arrest and prevents proliferation in murine SMG.** (A-C) Representative expression of 53BP1 (A,Aʹ), p21 (B) and Ki67 (C) in SMGs of uninjured mice or mice irradiated with single doses of 10 Gy or 15 Gy or multiple doses of 3×5 Gy and analysed 3 days after irradiation. Pink arrowheads indicate DNA damage foci (A′) or positive cells (B,C). A′ shows magnified views of the boxed areas in A to better show damage foci. Scale bars: 20 μm (A,B); 100 μm (C). (D-F) Enumeration of 53BP1^+^ foci/nucleus (D), p21^+^ cells (E) and Ki67^+^ cells (F) in SMGs collected from mice exposed to 0 Gy, 10 Gy, 15 Gy or 3×5 Gy irradiation and analysed 3 days after irradiation. Data were obtained from three fields of view from non-sequential sections from three to four mice per group and error bars represent the s.d. *P<0.05; **P<0.01; ***P<0.001 (one-way ANOVA with Tukey’s *Q* post hoc test).

We next examined cellular damage in response to radiation. We first performed immunofluorescence analysis for 53BP1 (encoded by *Trp53bp1*) to analyse the extent of DNA damage, as it forms visible foci in nuclei following exposure to ionising radiation (Gupta et al., 2014; Shibata and Jeggo, 2020). Indeed, we have previously shown that 53BP1^+^ foci are evident in the nuclei of ductal cells of the murine SMG at 1 and 3 days post irradiation (McKendrick et al., 2023). Here, we found evidence of 53BP1^+^ foci in the nuclei of cells in all three radiation regimes (Fig. 3A,A′), and the numbers of foci were significantly elevated compared to those in non-irradiated controls, in which there were no foci evident (Fig. 3D), consistent with previous findings for 10 and 15 Gy irradiation (Marmary et al., 2016; McKendrick et al., 2023). Although we found that the expression of *Cdkn1a* (encoding p21) was unchanged following radiation (Fig. 1C), we found that the numbers of p21^+^ cells in the irradiated tissues were significantly higher than those in non-irradiated controls (Fig. 3B,E). In contrast, we found that the numbers of proliferating cells, marked by Ki67 (encoded by *Mki67*), were significantly reduced following radiation, irrespective of the regime used (Fig. 3C,F). Overall, these findings demonstrate cellular damage caused by irradiation, leading to cell cycle changes.

**Fig. 4.**
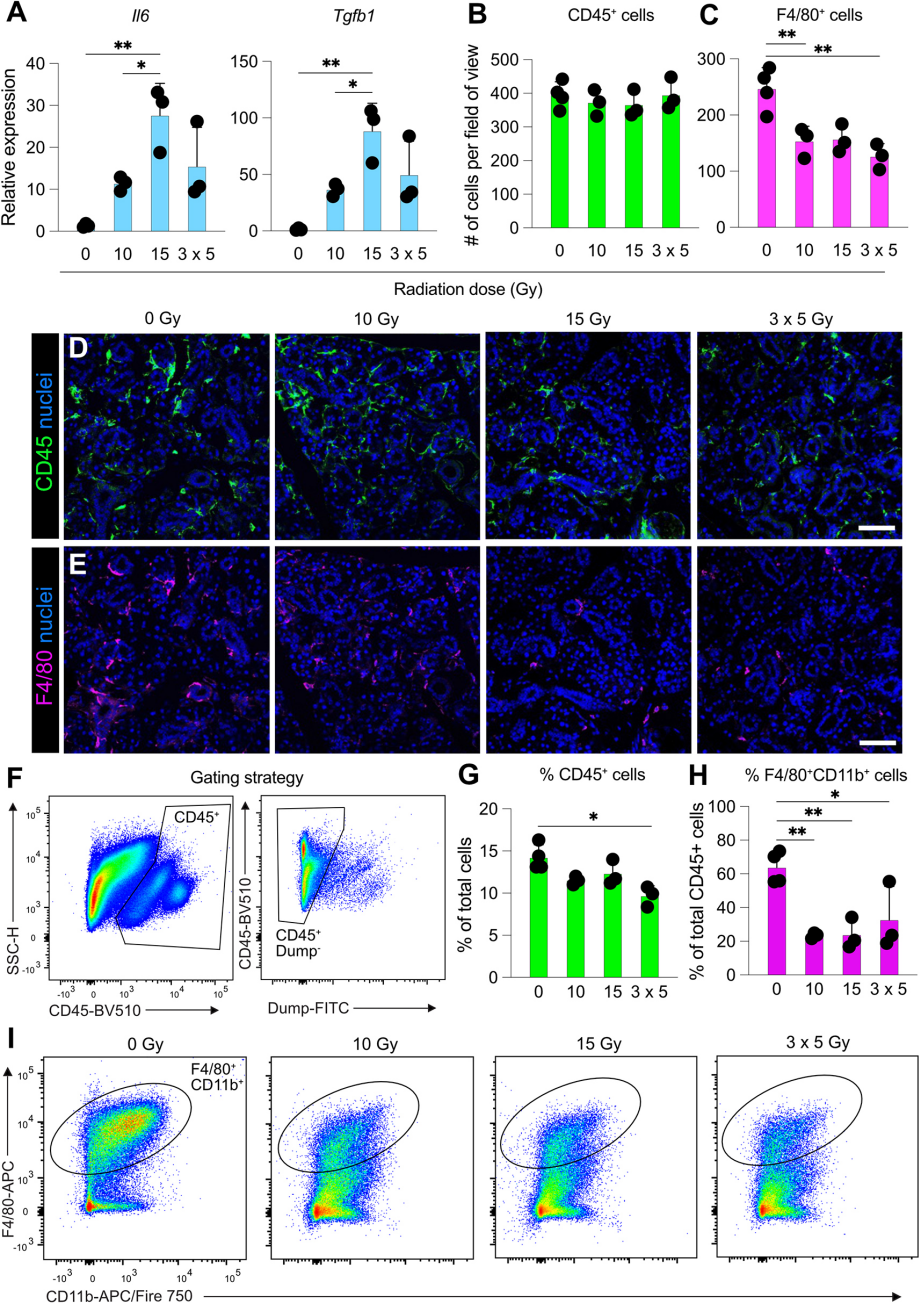
**Radiation injury induces expression of inflammatory cytokines and depletes tissue-resident SMG macrophages.** (A) qPCR analysis of *Il6* and *Tgfb1* mRNA in total SMG tissue at 3 days following radiation-induced injury. Data are normalised to mRNA levels in unirradiated (0 Gy) SMG tissue. (B,C) Enumeration of CD45^+^ (B) and F4/80^+^ (C) cells in SMGs collected from mice exposed to 0 Gy, 10 Gy, 15 Gy or 3×5 Gy irradiation and analysed 3 days after irradiation. Data were obtained from three fields of view from non-sequential sections. (D,E) Representative expression of CD45 (D) and F4/80 (E) obtained from SMGs of uninjured mice or mice irradiated with single doses of 10 Gy or 15 Gy or multiple doses of 3×5 Gy and analysed 3 days after irradiation. Scale bars: 100 μm. (F) Gating strategy for the identification of F4/80^+^ CD11b^+^ macrophages in the murine SMG. SSC-H, single scatter height. Dump-FITC consists of SiglecF^+^ cells, Ly6G^+^ cells and dead (SYTOX Green^+^) cells. (G,H) Enumeration of CD45^+^ cells as a percentage of total live cells (G) and F4/80^+^ cells as a percentage of CD45^+^ leukocytes (H) by flow cytometry in SMGs collected from mice exposed to 0 Gy, 10 Gy, 15 Gy or 3×5 Gy irradiation and analysed 3 days after irradiation. (I) Representative expression of CD11b and F4/80 in CD45^+^ leukocytes from SMGs at 3 days post radiation or from unirradiated mice (0 Gy). Data in A-C,G,H are from three to four mice per group and error bars represent the s.d. *P<0.05; **P<0.01 (one-way ANOVA with Tukey's *Q* post hoc test).

Radiation injury leads to inflammation and immune cell influx in numerous tissues (Cheng et al., 2021; Li et al., 2020; Nolan et al., 2022; Meziani et al., 2018; Groves et al., 2023). Conversely, tissue-resident immune cell populations and their niche can be altered following radiation injury (Groves et al., 2023; Hudson et al., 2023; Soysa et al., 2019; Venkateswaran et al., 2019). Although these changes can be beneficial if at the site of the tumour (Shinde-Jadhav et al., 2021), they can lead to negative effects on healthy surrounding tissues that are inadvertently irradiated during therapy. We found that radiation induced expression of the genes encoding the cytokines IL-6 (*Il6*) and TGFβ (*Tgfb1*) in whole SMGs (Fig. 4A). Sustained expression of IL-6 has been associated with salivary gland senescence and long-term hypofunction (Marmary et al., 2016), so the early upregulation seen in this study is not surprising and sustained expression could be causative in the longer-term dysfunction described in this previously published study. Furthermore, TGFβ plays a pivotal role in fibrosis after radiation injury (Dai et al., 2019) and accelerates the DNA damage response in epithelial cells *in vitro* via SMAD signalling (Lee et al., 2016). Interestingly, although significant upregulation of *Il6* and *Tgfb1* was observed in response to a single dose of 15 Gy radiation, this was not the case in the fractionated group, suggesting that low-dose-sustained injury does not have such a significant effect on cytokine expression, at least at this early timepoint after injury (Fig. 4A).

Finally, we have recently demonstrated the crucial role that macrophages play in salivary gland regeneration after irradiation injury (McKendrick et al., 2023). In this study, we examined the number of CD45 (encoded by *Ptprc*)-positive cells (i.e. total leukocytes) and macrophages at 3 days after radiation. We have previously shown that macrophages are the most abundant leukocyte in the salivary gland and that they express high levels of F4/80 (encoded by *Adgre1*) and medium to high levels of CD11b (encoded by *Itgam*) (McKendrick et al., 2023). Thus, we used these two markers to analyse macrophages in the current study, using both immunofluorescence staining coupled with confocal microscopy and flow cytometry. Although the number of total CD45^+^ leukocytes, measured by immunofluorescence analysis, was unchanged between unirradiated and radiated SMGs (Fig. 4B,D), the number of F4/80^+^ macrophages was significantly reduced after radiation in all regimes examined (Fig. 4C,E). This is in line with our previous findings that total SMG macrophages are significantly reduced at 3 days after 10 Gy targeted γ-irradiation, but that this loss is recovered by 7 days (McKendrick et al., 2023). This is likely due to the observed loss of a proliferative subpopulation of macrophages, which is eventually recovered (McKendrick et al., 2023). In order to explore this further, we confirmed this result by flow cytometry (Fig. 4F-I). The gating strategy for flow cytometry is shown in Fig. 4F. Here, we found that the number of CD45^+^ cells (as a percentage of total live cells) was significantly reduced with 3×5 Gy irradiation, but not with 10 or 15 Gy irradiation (Fig. 4G). However, we found that the total number of CD11b^+^ F4/80^+^ macrophages was significantly reduced following irradiation in all regimes (Fig. 4H,I), in line with results from immunofluorescence analyses.

## DISCUSSION

There is currently no established radiation regime to model radiotherapy-induced salivary gland injury in mouse models, and approaches range from low, single doses to large, fractionated doses. Here, we compared three different radiation regimes in order to directly compare the early injury that is induced, and show that although there are some subtle differences in different regimes, all approaches elicit similar levels of epithelial disruption, DNA damage and changes in cell cycle, and alterations to tissue-resident macrophages.

It is well established that off-target radiation causes a decline in saliva production (Eisbruch et al., 2001; Henson et al., 1999) changes in saliva consistency (Winter et al., 2021; Funegård et al., 1994; Makkonen et al., 1986), acinar cell loss, ductal metaplasia and fibrosis (Sullivan et al., 2005) in patients with head and neck cancer undergoing radiotherapy. However, it is challenging to explore the very early effects of radiation injury in human biopsies as material is scarce and the confounding factors of human studies make real data difficult to interpret. Thus, animal models provide a valuable resource to understand the cellular effects.

In this study, we observed transcriptional changes indicative of DNA damage in all three radiation regimes. We found elevated *Bax* expression at 3 days post irradiation, in line with previous studies that describe elevation of *Bax* expression at 3, 24 and 48 h post irradiation (Uchida et al., 2022), and elevation of *Bax* and *Puma* (also known as *Bbc3*) expression at 4 and 8 h post irradiation (Avila et al., 2009). However, these data are at odds with previous histological analysis of irradiated rat and mouse SMGs at 48 h post irradiation, which did not reveal detectable apoptosis (Marmary et al., 2016; Choi et al., 2009). This suggests that although the tissue exhibits DNA damage, either it does not undergo cell death via apoptosis, or the window of apoptosis is missed in these studies. Although apoptosis has been well described in the developing embryonic salivary gland (Teshima et al., 2016), reports on protein levels in the adult salivary gland after irradiation are scarce, with some exceptions (Ren et al., 2022; Gilman et al., 2019). However, we find that targeted radiation to the murine SMG leads to DNA damage foci, in line with previous reports in mice (Marmary et al., 2016; Ren et al., 2022; Uchida et al., 2022; Chang et al., 2019; Varghese et al., 2018). Given that radiation also causes DNA damage (marked by γH2AX and/or 53BP1 foci) in salivary gland cells *in vitro* (Uchida et al., 2022; Chang et al., 2019; Affandi et al., 2021) and salivary gland tissues *ex vivo* (Song et al., 2021), we speculate that this is a direct effect on the cells. Indeed, lymphocytes exhibit 53BP1 and γH2AX foci with increasing doses of irradiation (Sorokina et al., 2013). It is interesting that DNA damage is readily observed but apoptosis is not evident; this may mean that the DNA damage is not in fact leading to cell death and apoptosis. This phenomenon has been demonstrated in mammary gland and hair follicles, where stem cells exhibit DNA damage but are resistant to apoptosis, and instead exhibit enhanced DNA repair activity and continue to proliferate (Chang et al., 2015; Insinga et al., 2013; Sotiropoulou et al., 2010).

It has been proposed that the mechanism of action for the observed acinar cell loss following radiation is due to sublethal DNA damage, which manifests over time and finally becomes lethal at a delayed phase, resulting in acinar cell loss upon proliferation and replacement (Nagler, 2002). Given that we saw evidence of DNA damage in all three radiation regimes, coupled with the evident loss of AQP5 intensity, as previously described (Uchida et al., 2022; Choi et al., 2009), and reduction in acinar cell diameter, we propose that all three models tested here are capable of recapitulating the observed acinar cell loss in human salivary glands (Nam et al., 2016). Furthermore, we saw a reduction in the expression of *Aqp5* after 3 days, similar to what has been previously reported at 3 h post irradiation (Uchida et al., 2022). However, the previous study found that expression recovered by 24 h, whereas we observed that expression remained reduced at 3 days post irradiation across all three radiation regimes. Furthermore, we found that the expression of E-cadherin was not changed following irradiation, as has been previously described (Uchida et al., 2022). Although expression of *Cdkn1a*, the gene encoding p21, was unchanged at 3 days post irradiation, we observed significantly more p21^+^ cells in the SMG following irradiation, irrespective of the radiation regime analysed.

To date, most studies in which inflammatory cell infiltrate or resident inflammatory cells have been reported in post-radiation salivary glands have relied on Hematoxylin- and Eosin-stained images (Nam et al., 2016; Muhvic-Urek et al., 2006; Kim et al., 2019). However, in order to gain an accurate representation of inflammation, immunofluorescence and/or flow cytometric analysis is necessary. We found that although total leukocyte numbers were largely unchanged following irradiation injury, there was a reduction in the number of tissue-resident (F4/80^+^ CD11b^+^) macrophages, in line with our previous findings (McKendrick et al., 2023). Furthermore, the expression of pro-inflammatory cytokines was upregulated, in line with previous reports (Uchida et al., 2022). However, we cannot rule out that this may be an indirect effect: for example, *Cxcl2*, encoding a pro-inflammatory cytokine produced by macrophages, is upregulated in both oral tissues and SMGs after irradiation (Uchida et al., 2022; Shen et al., 2018). Moreover, bystander effects have been reported in non-irradiated contralateral salivary glands (Uchida et al., 2022), suggesting that irradiation injury also has systemic, long-range effects on salivary gland. This could explain why patients lose significant salivary gland function even when radiation is targeted on one side. We have previously shown that exposure to a single dose of 10 Gy γ-irradiation led to a substantial reduction in a proliferating subset of tissue-resident macrophages after 3 days (McKendrick et al., 2023). In all three radiation regimes tested in this study, we found a significant reduction in total macrophage numbers, which implies that exposure to either a single dose or multiple fractionated doses has a profound effect on tissue-resident macrophages.

In summary, we have demonstrated that, although there are subtle differences, the three radiation regimes analysed here are comparable in terms of DNA damage, cellular changes and macrophage abundance, and all three regimes mirror the early effects of radiotherapy injury. Furthermore, in order to induce injury in mouse salivary glands, most researchers will anaesthetise mice to immobilise them and directly irradiate the neck area only. However, a recent study has reported that repeated administration of ketamine affects salivary gland secretion and interferes with the reported loss following irradiation injury (Uchida et al., 2022). Thus, a model that involves minimum anaesthesia but recapitulates the injury evident in humans is both the most refined method and likely the most scientifically robust. However, we recognise that there will be differences in the source of ionising radiation and, as such, studies using X-rays may differ from those using γ-irradiation. That said, recent evidence suggests that γ-irradiation and X-ray irradiation elicit comparable effects in a model of bone marrow depletion for reconstitution (Wittenborn et al., 2021). Comparative research is required to ascertain whether this is also the case in salivary gland radiation injury. However, overall and based on the data presented, we propose that the lowest single dose tested here (10 Gy) is scientifically sound and the most appropriate model for future studies into acute cellular salivary gland damage.

### Limitations of the study

Although this study is informative, it has some caveats that should be recognised. Firstly, we only analysed SMGs from female mice; however, it is becomingly increasingly evident that sexual dimorphism exists in murine salivary gland structure and transcriptome (Mukaibo et al., 2019; Pinkstaff, 1998; Chai et al., 1993; Brown et al., 2020). Indeed, we have recently shown that there are subtle differences in immune cell populations between male and female mouse SMGs (McKendrick et al., 2023). Thus, male mice may exhibit subtle differences to female mice in response to radiation. This is not uncommon: male and female mice respond differently to radiation-induced arthrofibrosis (Rodman et al., 2022), lung toxicity (Bilal et al., 2014) and memory impairment (Perez et al., 2018); and male and female patients differ in sensitivity to irradiation-induced heart toxicity (Snyder et al., 2012) and laryngeal/pharyngeal side effects (Page et al., 2018). However, numerous studies, including our own, have used both male and female mice in salivary gland regeneration and have seen equivalent results (May et al., 2018; Emmerson et al., 2018; McKendrick et al., 2023); thus, we predict that male mice would also elicit comparable responses to the different regimes of irradiation tested here.

Secondly, we only assessed one timepoint following radiation in this study. However, we have previously shown that samples at 3 days post irradiation exhibit hallmarks of injury, including the expression of genes associated with apoptosis, loss of functional proteins, initiation of a DNA damage response and changes in macrophage proliferation (Emmerson et al., 2018; McKendrick et al., 2023); thus, this is a timepoint of significant interest. However, we cannot rule out the possibility that early and late effects are not differentially regulated across the three regimes tested. For example, following thoracic radiotherapy, fibrosis is not typically observed until 4-6 months post irradiation (Grundmann et al., 2009; Liu et al., 2018; De la Cal et al., 2012), and the extent of lung fibrosis increases as the dose of radiation increases (Rosen et al., 2001). Importantly, TGFβ signalling is a crucial mediator of the fibrotic response (reviewed by Meng et al. 2016; Frangogiannis 2020). However, it is becoming evident that expression of TGFβ in tissues including the lung and intestine varies depending on the dose and duration of radiation (Meyer et al., 2017b). Although the role of TGFβ in radiation-induced salivary gland fibrosis is yet to be elucidated, TGFβ expression is elevated in human salivary glands at 3 to 156 months after radiotherapy, and in mouse salivary glands at 2 weeks after radiation injury (Anscher et al., 1995; Hakim et al., 2011; Spiegelberg et al., 2014). However, a recent study has demonstrated elevated expression of TGFβ following ductal ligation (Woods et al., 2015), another salivary gland injury model that aims to recapitulate the damage caused by a ductal blockage (i.e. chronic obstructive sialadenitis or salivary gland stones) and exhibits significant fibrosis. Elevated TGFβ is also evident in clinical samples of human chronic obstructive sialadenitis (Teymoortash et al., 2003; Kizu et al., 1996). Given these data, it seems likely that elevated salivary gland TGFβ expression is a hallmark and possible cause of tissue fibrosis, but it is not unique to radiation injury. Cellular senescence is another key component of radiation-induced salivary gland dysfunction, which is mediated, at least in part, by IL-6 signalling (Marmary et al., 2016), but does not become apparent until 8-12 weeks post radiation. Although we report that the acute injury phase of irradiation-induced salivary gland damage is comparable between single doses of 10 Gy and 15 Gy and a fractionated dose of 3×5 Gy and, as such, 10 Gy is the most refined model, we cannot rule out the possibility that the models may differ when exploring different stages of the repair process and, in particular, the roles of TGFβ and IL-6, and the development of fibrosis and accumulation of cellular senescence.

Finally, as radiation therapy becomes more targeted in human patients, it is important that radiation-induced changes are recapitulated in animal models. Thus, it is important to recognise and respond to changes in preclinical testing, as reviewed by Verhaegen et al. (2023). However, to date, there has been no evidence that the benefits of proton therapy outweigh the costs for patients with head and neck cancer, and in the UK and USA, the majority of patients are still treated with conventional photon therapy (https://www.england.nhs.uk/publication/proton-beam-therapy-for-head-and-neck-cancer-in-adults/; Blanchard et al., 2018; Leeman et al., 2017; Sio et al., 2016). Thus, in this study, we have explored and validated the acute cellular responses of an animal model that most closely represents the injury that humans experience, highlighting the most refined experimental radiation regime.

## MATERIALS AND METHODS

### Mouse studies

All procedures were approved by the UK Home Office and performed under Project Licence PB5FC9BD (Emmerson). For the purposes of this study, only female mice were used.

### Radiation-induced salivary gland injury

Twenty-week-old C57BL/6 mice were anaesthetised using 1 mg/kg medetomidine hydrochloride (Dormitor) and 75 mg/kg ketamine (Ketavet) prepared in 0.9% saline (Thermo Fisher Scientific). The necks of anaesthetised mice were irradiated using a single ^137^Cs source in a Shepherd Mark-I-68A irradiator (JL Shepherd & Associates), as previously described (Emmerson et al., 2018; McKendrick et al., 2023). Non-irradiated control mice were not anaesthetised. After a 20 min period of anaesthesia, mice were given 1 mg/kg of the reversal agent Antisedan (Vetoquinol UK) and were allowed to recover in cages with additional heat support before returning to normal housing. Mice were provided with soft diet and DietGel (ClearH_2_O) *ad libitum*. Mice were euthanised at 3 days post irradiation and SMGs collected for analysis.

### Tissue digestion

Individual SMGs (approximately 80 mg) were mechanically minced in 2 ml of RPMI-1640 (Gibco) containing 5% foetal calf serum (FCS; Sigma-Aldrich), 25 µl collagenase-II (23 mg/ml) (Sigma-Aldrich), 25 µl hyaluronidase (40 mg/ml) (Sigma-Aldrich) and 125 µl of 0.1 M CaCl_2_ using a GentleMACS machine (Miltenyi Biotec) using program A.01 in a GentleMACS C-tube. Tissues were incubated in a shaking incubator at 100 rpm at 37°C for 60 min and subsequently centrifuged at 400 ***g*** for 5 min at 4°C. The supernatant was discarded and the pellet was resuspended in 2 ml of RPMI-1640 containing 5% FCS. The solution was filtered through a 70 µm nylon mesh (Thermo Fisher Scientific) and centrifuged at 400 ***g*** for 5 min at 4°C. The cell pellet was resuspended in 1 ml of 1× red blood cell lysis buffer (Abcam) for 5 min on ice before centrifugation at 400 ***g*** for 5 min at 4°C. The cell pellet was resuspended in 1 ml of pre-warmed (37°C) trypsin containing 0.25% EDTA and incubated at 37°C for 5 min before trituration. This step was repeated three times until single cells were evident. The solution was centrifuged at 400 ***g*** for 5 min at 4°C. Then, the cell pellet was resuspended in 1 ml of FACS buffer [Hank's Balanced Salt Solution (Lonza) containing 1% bovine serum albumin (BSA; Sigma-Aldrich) and 2 mM EDTA (Sigma-Aldrich)] and passed through a 20 µm filter-capped 5 ml fluorescence-activated cell sorting (FACS) tube (Falcon) using a 5 ml syringe with a 25G needle.

### Flow cytometry and FACS

Equal numbers of cells were resuspended in 100 µl of FACS buffer. Cells were stained with 1:1000 anti-CD16/32 (2.4G2; Biolegend) for 15 min at 4°C to reduce non-specific antibody binding to receptors for IgG. Cells were subsequently stained with conjugated antibodies (Table S1) for 60 min at 4°C in the dark. Samples were washed with FACS buffer and centrifuged at 400 ***g*** for 5 min at 4°C before resuspension in 300 µl of FACS buffer. Single stain controls were prepared using OneComp Beads (Thermo Fisher Scientific). Fluorescence minus one (FMO) controls were prepared using cells. SYTOX Green (Thermo Fisher Scientific) was used as a dead cell marker. Samples were analysed using an LSR II flow cytometer (BD Biosciences). Data were analysed using FlowJo software (v9). The dump gate (FITC) comprised SiglecF^+^ cells, Ly6G^+^ cells and dead (SYTOX Green^+^) cells.

### Tissue processing for histology

SMGs were fixed for 6-8 h in 4% paraformaldehyde (Thermo Fisher Scientific) at room temperature with constant mixing, followed by three washes with phosphate-buffered saline (PBS; Merck). After fixation, SMGs were processed for the generation of frozen sections by incubating in increasing concentrations of sucrose (10% and 30%) before embedding in OCT (Leica). 12 µm sections were cut using a cryostat (Leica) and stored at −20°C.

### Immunofluorescence analysis

Immunofluorescence analysis of tissue sections has been previously described (Emmerson et al., 2018). In brief, tissues were fixed with 4% paraformaldehyde if not previously fixed, and permeabilised with ice-cold acetone/methanol (1:1) for 1 min. Tissues were blocked for 2 h at room temperature with 5% BSA, 5% donkey serum (Merck) and 0.01% Tween 20 in PBS. Salivary glands were incubated with primary antibodies overnight at 4°C. Antibodies are listed in Table S2. Antibodies were detected using donkey Cy2-, Cy3- or Cy5-conjugated secondary Fab fragment antibodies (Jackson Laboratories, 711-546-152, 712-166-150, 705-166-147 and 712-546-153; 1:300), nuclei were stained using Hoechst 33342 (1:1000, Sigma-Aldrich), and slides were mounted using Prolong Gold anti-fade mounting medium. Fluorescence was analysed using a Leica SP8 confocal microscope and ImageJ software (National Institutes of Health).

### Histological analysis and cell counts

For immunofluorescence analysis, cells positively stained for markers were counted using ImageJ. Three random fields of view per sample were acquired on a Leica SP8 microscope at 25× or 40× magnification (Nyquist). Images were run through an ImageJ cell counting macro either as single images or, if the file was a *z*-stack, using the middle image of the stack. Using the macro, images were split into individual channels and the appropriate channel was extracted. Positive cells, such as macrophages, were thresholded and counted according to their size using the ‘Analyse particles’ command. As a quality control, an output file was saved where the counted macrophages were highlighted in green and could be manually checked and confirmed. This macro has previously been published in McKendrick et al. (2023). Fluorescence intensity was measured in ImageJ using the ‘Threshold’ function. Acinar cell size was measured as previously described (Lombaert et al., 2020) using the Image J ‘Measure’ function.

### Quantitative PCR analysis

RNA was isolated from a piece (∼3 mm^3^) of whole SMG tissue by sonicating in lysis buffer from the RNAqueous Micro Kit (Life Technologies) and following the manufacturer's instructions. Total RNA was DNase treated (Life Technologies) prior to cDNA synthesis (First Strand Synthesis Kit, Thermo Fisher Scientific). Quantitative PCR (qPCR) with SYBR Green was performed using 5 ng cDNA and primers [designed using Primer3 (https://www.primer3plus.com/index.html) and Beacon Designer software (https://www.premierbiosoft.com/molecular_beacons/) or described on PrimerBank (http://pga.mgh.harvard.edu/primerbank/)]. Primer sequences are listed in Table S3. Melt curves and primer efficiency were determined as previously described (Hoffman et al., 2002)*.* Gene expression was normalised to that of the housekeeping gene *Gapdh* and to a selected unirradiated control mouse sample. Reactions were run in triplicate.

### Statistical tests

Normal distribution was assessed using the D'Agostino–Pearson omnibus test. Data were analysed for statistical significance using one-way ANOVA (multiple groups) with post hoc testing performed using Tukey’s *Q* test (GraphPad Prism). For multiple testing, we used a false discovery rate of 0.05. All graphs show the mean±standard deviation (s.d.) as indicated in the figure legends. Statistical tests used for each experiment are also indicated in all figure legends.

## Supplementary Material

10.1242/dmm.050733_sup1 Supplementary information
