# Supplementary material for: A single dose of radiation elicits comparable acute salivary gland injury to fractionated radiation
Source: Dis Model Mech. 2024 Aug 22;17(8):dmm050733. doi: 10.1242/dmm.050733 (PMC11361643; doi:10.1242/dmm.050733)
Supplement: Supplementary information [file dmm-17-050733-s1.pdf]

Table S1. Antibodies used for flow cytometry

Antibody	Clone	Supplier	Cat #	Dilution	RRID
Rat CD11b APC/Fire 750	M1/70	Biolegend	101262	1:200	AB_2572122
Rat CD45 BV510	30-F11	Biolegend	103138	1:200	AB_2563061
Rat F4/80 APC	BM8	Invitrogen	17-4801-82	1:200	AB_2784648
Rat Ly6G FITC	1A8	Biolegend	127606	1:200	AB_1236494
Mouse SiglecF FITC	REA798	Miltenyi	130-112-178	1:200	n/a

Table S2. Primary antibodies used for immunofluorescent staining

Antibody	Clone	Species	Supplier	Cat #	Dilution	RRID
AQP5[w]		Rabbit	Millipore	AB3559	1:200	AB_2141915
E-Cadherin	ECCD2	Rat	Life Technologies	13-1900	1:300	AB_2533005
CD31		Goat	BioTechne	AF3628	1:300	AB_2161028
CD45		Goat	R&D Systems	AF114	1:200	AB_442146
F4/80	A3-1	Rat	Abcam	ab6640	1:200	AB_1140040
Ki67	SolA15	Rat	Invitrogen	14-5698-82	1:200	AB_10854564
p21	EPR18021	Rabbit	Abcam	ab188224	1:100	AB_2734729
53BP1		Rabbit	Novus	NB100-304	1:1200	AB_1659863

ω This antibody has been discontinued

Disease Models & Mechanisms • Supplementary information

Table S3. Primer sequences used for qPCR

Gene	Forward primer	Reverse primer
Gapdh	AGGTCGGTGTGAACGGATTTG	TGTAGACCATGTAGTTGAGGTCA
Aqp5	TCTACTTCTACTTGCTTTTCCCCTCCTC	CGATGGTCTTCTTCCGCTCCTCTC
Bax	TGAAGACAGGGGCCTTTTTG	AATTCGCCGGAGACACTCG
Ccnd1	CATCCATGCGGAAAATCGTGG	AAGACCTCCTCTTCGCACTTC
Cdkn1a	CCCCCAATCGCAAGGATTCTT	CTTGGTTCGGTGGGTCTGTC
Il6	CCGGAGAGGAGACTTCACAG	TCCACGATTTCCCAGAGAAC
Tgfb1	CTCCCGTGGCTTCTAGTGC	GCCTTAGTTTGGACAGGATCTG
